# Testing the Limits of Skill Transfer for Scrabble Experts in Behavior and Brain

**DOI:** 10.3389/fnhum.2016.00564

**Published:** 2016-11-09

**Authors:** Sophia van Hees, Penny M. Pexman, Ian S. Hargreaves, Lenka Zdrazilova, Jessie M. Hart, Kaia Myers-Stewart, Filomeno Cortese, Andrea B. Protzner

**Affiliations:** ^1^Department of Psychology, University of CalgaryCalgary, AB, Canada; ^2^Hotchkiss Brain Institute, University of CalgaryCalgary, AB, Canada; ^3^Seaman Family Magnetic Resonance Research Centre, University of CalgaryCalgary, AB, Canada

**Keywords:** Scrabble, expertise, far transfer, fMRI, ERP

## Abstract

We investigated transfer of the skills developed by competitive Scrabble players. Previous studies reported superior performance for Scrabble experts on the lexical decision task (LDT), suggesting near transfer of Scrabble skills. Here we investigated the potential for far transfer to a symbol decision task (SDT); in particular, transfer of enhanced long-term working memory for vertically presented stimuli. Our behavioral results showed no evidence for far transfer. Despite years of intensive practice, Scrabble experts were no faster and no more accurate than controls in the SDT. However, our fMRI and EEG data from the SDT suggest that the neural repertoire that Scrabble experts develop supports task performance even outside of the practiced domain, in a non-linguistic context. The regions engaged during the SDT were different across groups: controls engaged temporal-frontal regions, whereas Scrabble experts engaged posterior visual and temporal-parietal regions. In Scrabble experts, activity related to Scrabble skill (anagramming scores) included regions associated with visual-spatial processing and long-term working memory, and overlapped with regions previously shown to be associated with Scrabble expertise in the near transfer task (LDT). Analysis of source waveforms within these regions showed that participants with higher anagramming scores had larger P300 amplitudes, potentially reflecting greater working memory capacity, or less variability in the participants who performed the task more efficiently. Thus, the neuroimaging results provide evidence of brain transfer in the absence of behavioral transfer, providing new clues about the consequences of long-term training associated with competitive Scrabble expertise.

## Introduction

Scrabble is a popular board game in which players form words from an evolving set of seven tiles that they draw randomly. Players place tiles in horizontal or vertical strings on a game board, and after the first turn, new tiles must attach to at least one tile already played. Players accumulate points with each successful play, where the number of points are determined by the value of the tiles in each new word with bonus modifiers from the board itself. In the present work, we focus on the competitive version of the game, where strict time limits are imposed on game play, and penalties are exacted if players are caught playing “phonies” (non-words). Training for competitive Scrabble involves extensive rehearsal of word lists, anagramming, and considerable time spent in game play. Scrabble experts gain experience viewing and manipulating letters and words in both vertical and horizontal orientations (Fatsis, 2002). Much like chess, an official rating system quantifies Scrabble expertise, and players' ratings are correlated with the number of years spent training (Halpern and Wai, 2007; Tuffiash et al., 2007). Furthermore, the enhanced skills of competitive players are attributed to their training activities and competitive play, rather than pre-existing differences in vocabulary, visuospatial, or other skills (Halpern and Wai, 2007; Tuffiash et al., 2007).

Previous studies investigating expertise have shown that the enhanced performance of experts is supported by the development of unique knowledge structures, which create strategic or cognitive advantages in experts compared to non-experts (Bilalić et al., 2010; Harel et al., 2013; Chang, 2014). According to Tuffiash et al. (2007) *anagrammatic word-identification skill hypothesis*, the strategic/cognitive advantage developed during Scrabble training involves altered lexical processing: to efficiently identify written words, Scrabble experts rely on visual orthographic word knowledge and anagramming skill. Other aspects of word knowledge, such as phonology and semantic information, are de-emphasized. Tuffiash et al. further suggested that long-term working memory supports anagramming skills, such that Scrabble experts “acquire domain-specific representations to support effective encoding and access from long-term memory as a form of working memory” (p. 131). This conceptualization of working memory aligns with the skilled memory theory proposed by Ericsson and Kintsch (1995), which suggests that working memory incorporates skilled use of storage in long-term memory, such that experts show enhanced access to long-term memory via retrieval cues in working memory. Thus, the component processes in trained Scrabble include aspects of long-term working memory and visual orthographic processing (Tuffiash et al., 2007; Toma et al., 2014). A closely related fMRI study conducted by our group (Protzner et al., 2016) investigated the neural mechanisms underlying lexical processing in Scrabble experts using a visual lexical decision task. Consistent with the anagrammatic word-identification skill hypothesis, experts made use of brain regions not generally associated with meaning retrieval in visual word recognition, but rather those associated with working memory (e.g., superior parietal cortex) and visual perception (e.g., extensive activation in visual cortex).

Few studies have examined *transfer* of enhanced skill in the context of expertise (e.g., Green and Bavelier, 2003; Bidelman et al., 2011; Bartlett et al., 2013; Fauvel et al., 2013; Angelone et al., 2016), yet this is an interesting and important question. That is, it is important that we understand how specific experiences can shape the brain and affect behavior in other contexts. The concept of transfer in complex cognitive skills was addressed by Thorndike and Woodworth (1901) in their “Identical Elements” theory. This theory proposes that transfer of learning depends on the amount of similarity between the learning and transfer task, where the magnitude of transfer increases with greater amounts of overlap between tasks. Using this framework, it is possible to distinguish near transfer from far transfer based on relative task similarity (e.g., Barnett and Ceci, 2002; Schunk, 2004). Near transfer occurs when training improves performance on an untrained but similar task, for which there is strong overlap in task demands to the trained task. In contrast, far transfer occurs when training improves performance on an untrained and dissimilar task, for which there is little overlap in measured constructs.

The potential for *near* transfer of Scrabble skills has been tested in studies involving the standard word recognition task, lexical decision (LDT; Halpern and Wai, 2007; Hargreaves et al., 2012; Protzner et al., 2016). As in competitive Scrabble, LDT requires that participants work with letter strings and distinguish words from non-words. In the LDT, however, words and non-words are presented one string at a time on a computer screen. Further, while Scrabble requires that players create words that maximize point scores from randomly selected letters, LDT does not. In LDT, Scrabble expertise is associated with faster responses (Halpern and Wai, 2007; Protzner et al., 2016), especially for stimuli presented vertically (Hargreaves et al., 2012). That is, while vertically presented strings are more difficult for all readers to process (Howell and Bryden, 1987), the vertical presentation disadvantage is attenuated for Scrabble experts. This finding was attributed to experience-driven flexibility in orthographic encoding (Hargreaves et al., 2012). Because Scrabble play involves experience with vertical word recognition, Scrabble players develop the ability to efficiently extract orthographic information even from vertically presented stimuli. It is not clear, however, whether this vertical fluency for Scrabble experts is limited to letter stimuli or whether it might transfer to non-letter visual stimuli.

To investigate this question of far transfer and to probe the limits of Scrabble expertise, we compared performance of Scrabble experts and controls in the symbol decision task (SDT). In the SDT, the letter strings of the LDT are replaced with unfamiliar symbols. As in the LDT, there is a binary decision involved (participants distinguish strings of all unique symbols from strings with one symbol presented twice), and importantly, the SDT requires judgments about both horizontally—and vertically-presented visual strings. We used this task to test whether the vertical fluency of Scrabble experts extends to unfamiliar visual symbols. We considered this task a test of far transfer because, while there are some similarities, the demands of this task are different than those of Scrabble or the LDT. The SDT does not require the same kind of access to long-term lexical memory that is involved in Scrabble and LDT. Instead, it likely relies on visual perception and working memory as participants hold visual symbols in mind and search for a match in each string. Whereas Scrabble and LDT require visual processing of familiar symbols, the SDT requires visual processing of unfamiliar symbols.

We tested for far transfer to the SDT in behavioral data to probe for potential performance differences, and in brain data with fMRI to probe for location differences, and with ERP source waveforms (drawing on regions identified in our fMRI analyses) to probe for timing differences. It is possible that reduced activity compared to controls may be associated with increased task efficiency in the Scrabble experts, in line with the intelligence literature (e.g., Haier et al., 1992; Jausovec and Jausovec, 2004). However, previous studies in the expertise literature suggest that functional reorganization and greater bilateral activity underlies expert performance (e.g., Bilalić et al., 2011, 2012; Guida et al., 2012; Proverbio et al., 2013). Thus, evidence for far transfer in the current study may include greater activity in bilateral visual-perceptual and long-term working memory regions for Scrabble experts compared to controls during the SDT. Analysis of ERP amplitudes within these regions may additionally identify group differences in finer-grained temporal information. Furthermore, to more directly link potential group differences to Scrabble expertise, we included analyses that were constrained to show only those similarities and differences between groups that were associated with a measure of Scrabble expertise: anagramming scores. Anagramming scores are correlated with official rankings in the North American Scrabble Players Association (NASPA) for Scrabble experts and, unlike NASPA rankings, it is possible to obtain anagramming scores for both Scrabble expert and control participants.

## Methods

### Participants

Participants were 21 competitive Scrabble players and 24 non-expert controls. Due to behavioral performance no greater than chance (two participants), or issues with data acquisition (three participants), five controls were excluded from the analysis. Additionally, one control participant was excluded from the EEG analyses, due to electrophysiological amplitudes greater than 2.5 standard deviations from the mean. Two Scrabble experts were also excluded due to information disclosed following data collection. One participant disclosed a history of severe head injury, and another a history of drug abuse and diagnosis of Attention Deficit Disorder. Following these exclusions, the data for 19 Scrabble experts (10 males), and 19 age-matched controls (9 males) were included in the analysis. All participants were right handed and there were no significant differences between groups in terms of age or years of education. The Scrabble experts ranged from 24 to 79 years of age (*M* = 57.2, *SD* = 18.05) and controls ranged from 24 to 83 years of age (*M* = 55.9, *SD* = 16.32). With respect to years of education, Scrabble experts ranged from 11 to 20 years (*M* = 16.61, *SD* = 3.00) and controls ranged from 12 to 20 years (*M* = 17.42, *SD* = 3.30). As expected, Scrabble experts reported that they spent a significantly greater number hours per week playing Scrabble (*M* = 8.29, *SD* = 5.79) than did controls (*M* = 0.89, *SD* = 3.20, *p* < 0.001). In contrast, there was no significant group difference in reported hours spent engaged in crosswords for Scrabble experts (*M* = 1.04, *SD* = 1.76), and controls (*M* = 1.55, *SD* = 3.17).

EEG and fMRI were collected in separate testing sessions. All 19 Scrabble experts and 18 of the 19 controls were included in the EEG analyses. A subset of 12 participants from each group also completed the fMRI component of the study (one control participant who completed the fMRI component was not included in the EEG analyses), and data from this subset of participants were also reported in our previous study (Protzner et al., 2016). Scrabble players were recruited through extensive advertising over a 1-year period at local and national Scrabble competitions held in the Calgary area. Control participants were recruited through community advertising. The study was approved by the University of Calgary Conjoint Faculties Research Ethics Board and the Conjoint Health Research Ethics Board, and all participants gave written informed consent prior to partaking in the study.

### Cognitive tests

All participants completed the following battery of cognitive assessments to assess potential generalized differences in cognitive function between groups: (1) the WAIS III Digit Symbol subtest (Wechsler, 1997), a measure of perceptual speed that involves matching symbols to a list of numbers with reference to nine digit-symbol pairs, (2) the North American Adult Reading Test, (NAART; Uttl, 2002), a vocabulary test involving pronunciation of irregularly spelled English words, (3) the Author Recognition Test (ART; Acheson et al., 2008), a test of print exposure requiring identification of real author names from a list of 130 names to ensure any group differences in linguistic processing were not due to differences in print exposure, (4) the Controlled Oral Word Association Test (COWAT; Spreen and Strauss, 1998), involving production of as many words as possible within 60 seconds using the orthographic categories of “F,” “A,” “S,” and “UN,” as well as the semantic category “animals,” and (5) anagramming skill, assessed using 51 anagrams presented on a computer (Tuffiash et al., 2007).

### Stimuli

Stimuli for the SDT were created using 26 non-letter symbols in Microsoft Word. Each stimulus string contained five symbols, with 216 strings containing two of the same symbol (match, or “yes” response, e.g., 
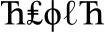
) and 144 strings containing all unique symbols (no-match, or “no” response, e.g., 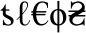). Items were divided into three lists, each consisting of 72 matches and 48 no-matches.

Although the focus of this paper is on *far transfer* to the SDT, we report behavioral data for the LDT to establish that the *near transfer* effects found in previous studies (Halpern and Wai, 2007; Hargreaves et al., 2012; Protzner et al., 2016) hold for our sample. Stimuli for the LDT were selected from a set of 432 words and 288 non-words, with similar characteristics to those used in the previous study by Hargreaves et al. (2012). These items were divided into three lists, each consisting of 144 words and 96 non-words. Across lists, word stimuli were matched for length, frequency, orthographic neighborhood, age of acquisition, and imageability. Word and non-word stimuli were matched for visual characteristics including orthographic Levenshtein distance, a measure quantifying the number of letter substitutions, insertions, or deletions required to change one word into another (Yarkoni et al., 2008) and word length. Word and non-word stimuli ranged between 4 and 8 letters (*M* = 5.22, *SD* = 1.14).

**Figure 1:**
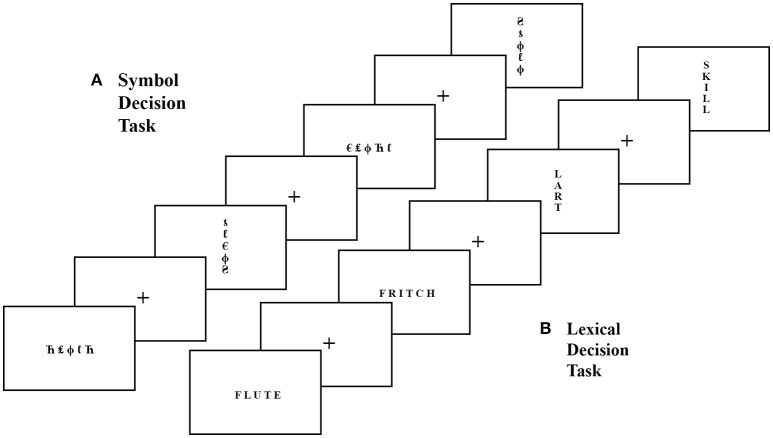
**Example stimuli and task structure for the Symbol Decision Task (SDT; A) and Lexical Decision Task (LDT; B)**. For the SDT, participants made yes/no decisions whether symbol strings, presented horizontally and vertically, contained two of the same symbol (match) or not (no-match), with a central fixation cross between each trial. For the LDT, participants made yes/no decisions whether letter strings, presented horizontally and vertically, were real words or non-words, with a central fixation cross between each trial.

For each task, stimuli from two of the three lists were used in the EEG session (counterbalanced across participants), and the third was used for the fMRI session. During each task, half the stimuli were presented horizontally, and half were presented vertically. Within each list, the order of stimuli varied randomly, and the orientation of presentation was counterbalanced across participants. For each trial, participants were instructed to provide a yes/no decision as to whether the stimulus was a match (for SDT) or contained a word (for LDT) as quickly and accurately as possible (see Figure 1).

### fMRI procedure

Stimuli were presented one at a time, back projected (Avotec, Inc., Stuart, FL, U.S.A), onto a MRI compatible screen using Presentation software version 16.0 (Neurobehavioural Systems, Inc., Berkeley, CA, U.S.A). Participants made yes/no decisions with their right hand using two buttons on an MR-compatible Lumina LSC-400B response pad (Cedrus, San Pedro, CA, U.S.A). Participants completed one stimulus list for each task; the list was presented over three runs for the LDT and two runs for the SDT. Each trial began with the presentation of a fixation cross for 500 ms, followed by the string for 2000 ms. Inter-trial intervals were jittered to increase the detectability of hemodynamic responses to trials (Birn et al., 2002), and were between 3500 and 7500 ms in duration (mean 5500 ms).

### EEG procedure

Stimuli were presented one at a time on a 24-inch monitor (HP lp2475w) using Presentation software version 16.1 (Neurobehavioural Systems, Inc., Berkeley, CA, U.S.A). Participants were seated approximately 80 cm from the computer screen and the visual angle of the SDT stimuli was 3.9 × 0.7°. For each trial, a central fixation cross was presented for jittered durations between 250 and 750 ms (mean 500 ms), followed by the stimulus. Participants made yes/no decisions with their right hand using the left and right buttons on a mouse. The string remained on the screen until a response was made, triggering the beginning of the next trial. Participants completed two stimulus lists for each task, with a short break between each list.

### fMRI data acquisition and pre-processing

Anatomical and functional MRI data were acquired on a 3T whole-body MR scanner (Discovery 750; GE Healthcare, Waukesha, WI, U.S.A.). Functional data were acquired in an interleaved order (TR = 2 sec; 37 slices, 220 mm FOV, 64 × 64 matrix, resulting in a voxel size of 3.75 × 3.75 × 3.40). Three-dimensional anatomical scans were acquired with higher spatial resolution (T1-weighted sequence, 236 slices, 256 mm FOV, 256 × 256 matrix, resulting in a voxel size of 1.0 × 1.0 × 1.0 mm).

For fMRI preprocessing, using SPM8 (http://www.fil.ion.ucl.ac.uk/spm/; Friston et al., 1995), time series data were spatially coregistered to correct for head motion by using a 3D Fourier transform interpolation. Functional data were corrected for artifacts via independent component analysis (ICA) within separate runs, as implemented in FSL/Melodic (Beckmann and Smith, 2004). Probabilistic ICA assumes that artifacts present in fMRI data follow a non-Gaussian distribution. It is a blind source separation technique that decomposes a two-dimensional data matrix (time by voxels) into a set of time courses with associated spatial maps, which jointly describe the temporal and spatial characteristics of statistically independent latent variables (source signals). Artifacts were identified and removed following the guidelines outlined by Beckmann and Smith (2004). Specifically, the ICA denoising procedure was performed by one of the authors (K.M.S), who identified artifactual components for each participant and each run based on combined information from the spatial distribution, time series, and spectral power distribution of ICA components. The most common characteristics of artifacts were focality of the spatial distribution, ratio of weights within ventricles vs. brain, ratio of weights along the outer edge of the brain vs. the rest of the brain, relative spectral power distribution within low-, medium—and high-frequency ranges, and presence of spikes within the time course. Identified noise components were subsequently removed from the data set (using the fsl_regfilt function from FSL). The data were then coregistered, segmented and normalized into MNI space using SPM8 default settings. Finally, default SPM smoothing was applied using 8 mm Gaussian kernel. Because we used a conservative approach in the identification of noise artifacts, voxel time series were further adjusted by regressing out motion parameters, white matter (WM) and CSF time series. For WM and CSF regression, one of the researchers (K.M.S) extracted time series from unsmoothed data within small ROIs in the corpus callosum and ventricles of each participant using FSLView. The templates from each participant were then converted to text files and loaded into FSL along with motion parameters in the same format. Once in FSL, the FEAT (fMRI Expert Analysis Tool) with FILM (FMRIB's Improved Linear Model) prewhitening was implemented to remove any remaining artifactual noise using a multiple regression, thus creating a noise reduced time series for each voxel.

### EEG data acquisition and pre-processing

EEG recordings were acquired in a dimly lit, quiet room. Continuous EEG was recorded from an EasyCap (10/20 positioning system) containing 64 active electrodes with Cz as reference using BrainVision actiCHamp system (Brain Products GmbH, Gilching, Germany). Data were digitized continuously at a 500 Hz sampling rate with a band pass of 0.05–100 Hz.

Raw data were bandpass filtered at 0.1–55 Hz and re-referenced to a common average reference. Artifact removal was performed on the continuous data using independent component analysis (ICA) as implemented in Brain Electrical Source Analysis (BESA) software package. Components carrying ocular artifacts, such as blinks, saccades, horizontal eye movements, or other muscle artifacts were removed. The continuous data were then segmented into epochs from 200 ms pre-stimulus onset to 1000 ms post-stimulus onset, and baseline corrected to the 200 ms pre-stimulus interval. The two blocks were combined to form grand averaged waveforms for each individual, which were then used to form grand averaged waveforms for each group.

### Behavioral analyses

To examine the possibility of far transfer, response latencies and accuracy for the SDT and LDT were analyzed with a 2 (task: SDT, LDT) × 2 (group: controls, Scrabble) × 2 (condition: match, no-match) × 2 (orientation: horizontal, vertical) repeated measures ANOVA.

### Image analyses

We next outline the image analyses performed, and the question tested in each case. All of these analyses were performed using partial least squares (PLS) analysis (http://www.rotman-baycrest.on.ca/index.php?section=345; McIntosh et al., 1996; Lobaugh et al., 2001; McIntosh et al., 2004), which is a multivariate technique that identifies groups of brain regions distributed over the entire brain that together co-vary with some aspect of the experimental design. Task PLS identifies large-scale patterns of brain activity, or latent variables (LVs), which highlight similarities or differences between participant groups and experimental conditions. Behavior PLS identifies large-scale patterns of brain-behavior correlations, or LVs, that highlight similarities or differences between participant groups and experimental conditions. PLS is most commonly used in a data-driven manner, where hypotheses about group and/or condition effects are not specified in advance. In addition to the data-driven version of PLS, we used the *non-rotated* version for hypothesis-driven analyses, in which a priori contrasts restricted the patterns derived (McIntosh and Lobaugh, 2004). Statistical assessment was performed using permutation tests for the LVs and bootstrap estimates of standard errors for the voxel saliences. For the current paper, we used 500 permutations, and 200 bootstraps. For both fMRI and EEG results, we designated a minimum bootstrap threshold of 2.8, corresponding to a 99.5% confidence interval, or a *p* < 0.005. In some analyses, we used higher bootstrap thresholds (corresponding to even lower *p*-values). For fMRI analyses, our minimum cluster size was 10 voxels. We describe task and behavior PLS in more detail in Supplementary Materials.

#### fMRI analysis 1 two-group hypothesis driven task PLS examining group differences in SDT

Using our fMRI data, we assessed whether there were group differences in the neural regions engaged during the SDT for all four conditions (i.e., horizontal match, vertical match, horizontal no-match, and vertical no-match).

#### fMRI analysis 2 two-group data-driven behavior PLS examining brain-anagramming score correlations in SDT

Using our fMRI data, we assessed whether there were group—and SDT-condition-dependent differences in the regions where brain activity during SDT correlated with individual differences in anagramming scores. This constrained our analysis to focus on group differences linked to Scrabble expertise.

#### fMRI analysis 3 two-group hypothesis-driven behavior PLS examining brain-anagramming score correlations in LDT and SDT

Using our fMRI data, we examined if there were commonalities amongst regions where brain activity during the four SDT conditions and four LDT conditions (i.e., horizontal word, vertical word, horizontal non-word, and vertical non-word) correlated with individual differences in anagramming scores across groups.

#### ERP source model analysis 1 two-group data-driven behavior PLS examining brain-anagramming score correlations in SDT

Drawing on the regions identified in fMRI Analysis 2 as positively correlating with anagramming scores and supporting SDT performance in both groups, we created source models. Within these sources, we examined potential group differences in brain-anagramming score correlations in the four SDT conditions. As before, we constrained our analysis to focus on timing that was associated with anagramming scores to ensure that any potential group differences were linked to Scrabble expertise.

#### ERP source model analysis 2 control group data-driven behavior PLS examining brain-anagramming score correlations in SDT

Drawing on the regions identified in fMRI Analysis 2 as positively correlating with anagramming scores and supporting SDT performance in controls, we created source models. Within these sources, we examined brain-anagramming score correlations for the four SDT conditions.

#### ERP source model analysis 3 scrabble group data-driven behavior PLS examining brain-anagramming score correlations in SDT

Drawing on the regions identified in fMRI Analysis 2 as positively correlating with anagramming scores and supporting SDT performance in Scrabble experts, we created source models. Within these sources, we examined brain-anagramming score correlations for the four SDT conditions.

### Source modeling for EEG data (used for ERP source analyses 1, 2, and 3)

Source modeling of the ERP data was performed using Brain Electrical Source Analysis (BESA version 6.0) software package (MEGIS Software, GmbH, Munich; Scherg and Berg, 1996), using a four-shell ellipsoidal head model with relative conductiveness of 0.33, 0.33, 0.0042, and 1 for the head, scalp, bone, and cerebrospinal fluid, respectively, and sizes of 85 mm (radius), 6 mm (thickness), 7 mm (thickness), and 1 mm (thickness). The time window from 0 to 1000 ms was selected from the grand averaged files for each group. From the group similarities LV of fMRI Analysis 2 (Behavior PLS Examining Brain-Anagramming Score Correlations in SDT), we chose the regions that had a common positive correlation with anagramming scores across all groups and conditions (i.e., the five regions with positive saliences in **Table 3**). Separate models were calculated for each group and each of the four SDT conditions that made a reliable contribution to the LV in the fMRI analyses. We initially fit all regions to the model, to identify the percent variance accounted for by each region. Next, we calculated a new model for each condition, where the region that accounted for the most variance was fit to the model first, followed by the region accounting for most of the remaining variance, until adding additional regions accounted for less than 2% of the remaining variance across groups/conditions. Using this method, four regions were fit in to the model (right medial temporal pole, followed by the right precentral gyrus, the left middle temporal gyrus, and the right temporal pole). Each source contained three orthogonal dipoles to account for all directions of current flow at the source location (tangential, radial, and anterior/posterior). Maintaining orthogonal constraints, the orientations of the tangential sources were then aligned with the maximum direction of activity and the regional sources were converted to single dipoles to calculate source waveforms from these regions for each participant for statistical analysis.

From the group differences LV of fMRI Analysis 2, separate models were created for each group, as different regions were identified in controls compared to Scrabble experts. For controls, data from each of the four conditions were fit in to the model. For Scrabble experts, data from the horizontal match condition were not used, as that condition did not make a reliable contribution to the LV (as indicated by the fact that the confidence interval crossed zero on the correlation bar graph, see **Figure 4**). For controls, we first fit the regions that had a common positive correlation with anagramming scores (i.e., the fifteen regions with positive saliences in **Table 4**) to identify the percent variance accounted for by each region. For Scrabble experts, we first fit the regions that had a common positive correlation with anagramming scores (i.e., the eleven regions with negative saliences in **Table 4**) to identify the percent variance accounted for by each region separately for each condition. Next, we calculated new models for each condition, where the regions that accounted for the most variance were fit in to the models first, followed by the region accounting for most of the remaining variance, until adding additional regions accounted for less than 2% of the remaining variance across conditions. Using this method, four regions were fit in to each model (Controls: the left lingual gyrus, followed by the right fusiform gyrus, the left mid cingulate cortex, and the left middle temporal gyrus; Scrabble: the left superior parietal lobe, followed by the left fusiform gyrus, the right thalamus, and the right middle temporal gyrus). Using the method described in the previous paragraph, these regional sources were then converted to dipole sources to calculate source waveforms from these regions for each participant for statistical analysis.

## Results

### Cognitive assessments

As illustrated in Table 1, Scrabble and control groups showed significant differences in cognitive assessment results only for Scrabble-related skills (verbal fluency and anagramming). NASPA ratings were positively correlated with anagramming scores for the Scrabble experts (*r* = 0.53, *p* = 0.01). No significant group differences were found in cognitive assessments that were not directly Scrabble-related (i.e., perceptual speed, vocabulary, and print exposure). A subset of the cognitive assessment results have been reported previously in our study examining near transfer for the 12 participants in each group who underwent fMRI scanning (Protzner et al., 2016).

**Table 1:** **Means (and Standard Deviations) of Cognitive Assessment Results in Each Group**.

	**Scrabble experts**	**Age-matched controls**
WAIS III Digit Symbol Speed	78.5 (19.8)	73.0 (16.2)
NAART Vocabulary	26.5 (6.0)	23.1 (7.9)
ART	29.3 (11.4)	27.5 (15.0)
COWAT Word Fluency: F	22.1 (5.7) ^**^	16.8 (5.3)
COWAT Word Fluency: A	20.1 (5.5) ^**^	13.5 (6.1)
COWAT Word Fluency: S	22.3 (6.0) ^*^	17.5 (5.7)
COWAT Word Fluency: UN	14.0 (5.5) ^*^	9.5 (3.7)
COWAT Word Fluency: Animal	24.5 (7.5) ^*^	20.3 (6.2)
Anagram Accuracy (%)	53.5 (15.0) ^***^	19.0 (9.7)
NASPA Rating	1209.9 (299.2)	NA

*WAIS, Wechsler Adult Intelligence Scale; NAART, North American Adult Reading Test; ART, Author Recognition Test; COWAT, Controlled Oral Word Association Test; NASPA, North American Scrabble Players Association*.

* *p <0.05*,

** *p <0.01*,

*** *p <0.001*.

### Behavioral results

The behavioral data from the fMRI and EEG sessions showed the same pattern of results. For the sake of parsimony, and as only a sub-set of participants completed the fMRI session, behavioral results from the EEG tasks are reported below. For each participant, trials with response latencies greater than 2.5 SDs from the mean of each condition were considered outliers and were excluded from the analyses (2.03% of SDT trials, and 2.54% of LDT trials). Incorrect responses were excluded from the latency analyses (8.66% of SDT trials, and 4.76% of LDT trials).

Response latencies and accuracy from both tasks were first analyzed together. The analysis of response latencies showed significant main effects of task, *F*_(1, 35)_ = 73.50, *p* < 0.001, $\eta_{\text{p}}^{2}$ = 0.68, condition, *F*_(1, 35)_ = 85.22, *p* < 0.001, $\eta_{\text{p}}^{2}$ = 0.71, and orientation, *F*_(1, 35)_ = 123.34, *p* < 0.001, $\eta_{\text{p}}^{2}$ = 0.78. That is, participants were slower to respond to stimuli in the SDT compared to the LDT, to no-match/non-word conditions compared to match/word conditions, and to vertical trials compared to horizontal trials. The interaction between group and task was not significant (*p* > 0.05).

The analysis of accuracy showed significant main effects of task, *F*_(1, 35)_ = 8.58, *p* < 0.001, $\eta_{\text{p}}^{2}$ = 0.20, condition, *F*_(1, 35)_ = 12.98, *p* = 0.001, $\eta_{\text{p}}^{2}$ = 0.27, and orientation, *F*_(1, 35)_ = 25.06, *p* < 0.001, $\eta_{\text{p}}^{2}$ = 0.42. That is, participants were less accurate in responding to stimuli in the SDT compared to the LDT, to no-match/non-word conditions compared to match/word conditions, and to vertical trials compared to horizontal trials. Again, the interaction between group and task was not significant (*p* > 0.05) so the behavioral data for the two tasks were next analyzed separately.

#### SDT response latencies

**Figure 2:**
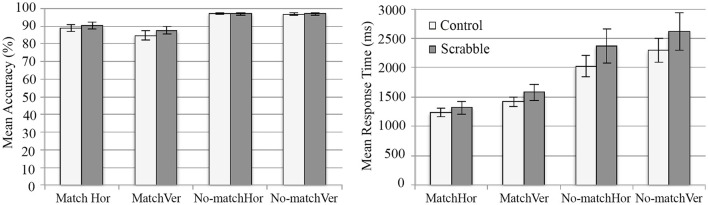
**Percent accuracy (left) and mean response times (right) for SDT match and no-match trails in horizontal and vertical orientations for the competitive Scrabble group and control group**. Hor, horizontal presentation; Ver, vertical presentation; SDT, Symbol Decision Task. Error bars indicate standard error.

There were significant main effects for condition, *F*_(1, 35)_ = 68.51, *p* < 0.001, $\eta_{\text{p}}^{2}$ = 0.66, and orientation, *F*_(1, 35)_ = 96.29, *p* < 0.001, $\eta_{\text{p}}^{2}$ = 0.73. Participants were slower to respond to no-match trials as compared to match trials, and were also slower to respond to trials presented vertically as compared to those presented horizontally (see Figure 2). There was no significant main effect of group, nor any significant interactions between group, condition, or orientation.

#### SDT accuracy

There were significant main effects for condition, *F*_(1, 35)_ = 55.69, *p* < 0.001, $\eta_{\text{p}}^{2}$ = 0.61, and orientation, *F*_(1, 35)_ = 7.93, *p* < 0.01, $\eta_{\text{p}}^{2}$ = 0.19, as well as a significant interaction between condition and orientation, *F*_(1, 35)_ = 4.91, *p* = 0.03, $\eta_{\text{p}}^{2}$ = 0.12. Participants were significantly less accurate for vertical match trials compared to horizontal match trials, *t*_(36)_ = 2.91, *p* < 0.01, whereas no significant difference in accuracy was found between orientations for no-match trials (*p* = 0.79) (see Figure 2). There was no significant main effect of group, nor any significant interactions between group, condition, or orientation.

#### LDT response latencies

**Figure 3:**
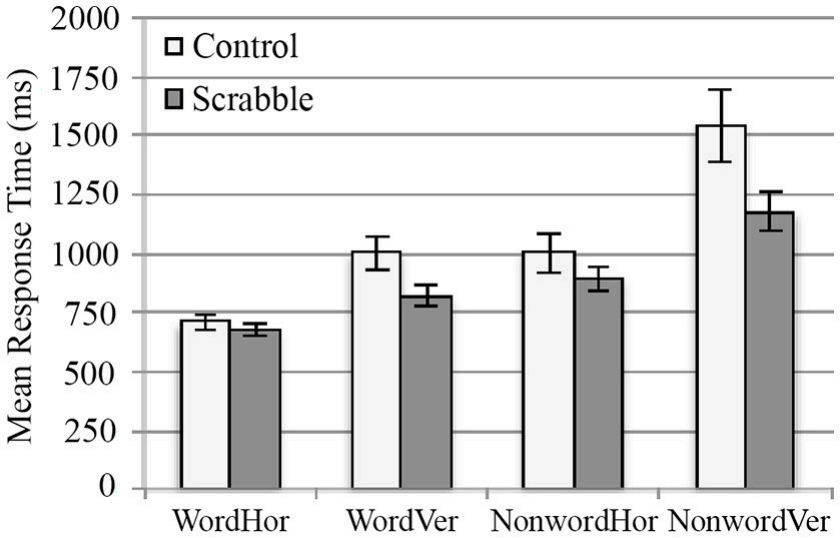
**Mean response times for LDT word and non-word trails in horizontal and vertical orientations for the competitive Scrabble group and control group**. Hor, horizontal presentation, Ver, vertical presentation, LDT, Lexical Decision Task. Error bars indicate standard error.

Analysis of response latencies in the LDT found the expected vertical fluency effect for Scrabble experts for both word and non-word stimuli. There was a significant three-way interaction of lexicality, orientation, and group, *F*_(1, 35)_ = 4.84, *p* = 0.03, $\eta_{\text{p}}^{2}$ = 0.12. Scrabble experts were significantly faster than controls at correctly responding to vertical words, *t*_(35)_ = 2.15, *p* = 0.038, and non-words, *t*_(35)_ = 2.10, *p* = 0.043 (see Figure 3). No significant differences were found between groups for horizontally presented items. There were too few error trials to warrant accuracy analyses for the LDT data.

### fMRI results

#### fMRI analysis 1 two-group hypothesis-driven task PLS examining group differences in SDT

**Figure 4:**
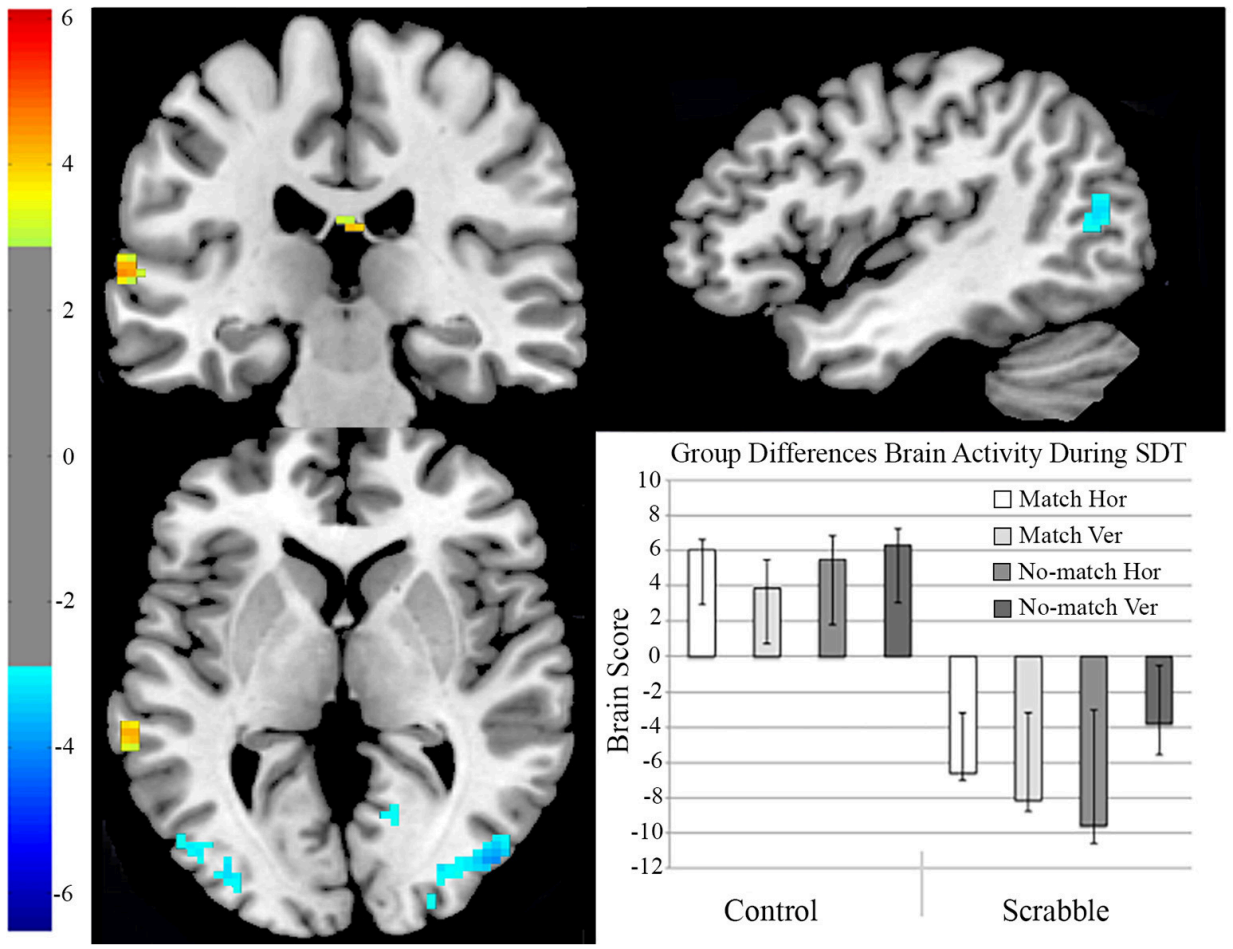
**fMRI analysis 1, two-group hypothesis-driven task PLS examining group differences in SDT**. On the brain images we illustrate regions with maximal activity differentiation between control and Scrabble groups during the SDT. The brain is displayed in 3-plane view according to neurological convention (L = L). Yellow regions represent increased activity for the control group, and blue regions represent increased activity for the Scrabble expert group. The brain scores bar graph captures the mean brain score for each condition in each group. The error bars indicate the 95% confidence intervals derived from bootstrap estimation. Hor, horizontal presentation; Ver, vertical presentation; SDT, Symbol Decision Task.

We first examined whether the Scrabble experts engaged different brain regions during the SDT as compared to matched controls. We found a significant group difference across all conditions of the SDT (*p* = 0.004, see Figure 4). For controls, regions engaged during SDT performance included the middle temporal, lingual, and superior medial gyri in the left hemisphere, and the medial temporal pole, precuneus, and cuneus in the right hemisphere. For the Scrabble experts, regions engaged during SDT performance included bilateral visual areas (lingual gyri, middle and superior occipital gyri), left hemisphere language regions and their right hemisphere homologs (e.g., fusiform, supramarginal, inferior frontal, middle/superior/inferior temporal gyri, and inferior parietal lobe), as well as middle and superior frontal gyri, and cingulate cortex (see Table 2 for a full list of regions).

**Table 2:** **fMRI Analysis 1, Non-rotated Task PLS Examining Group Differences in SDT**.

**Lag**	**x**	**y**	**z**	**BSR**	**Cluster size**	**Region**	**BA**
**LEFT LATERALIZED POSITIVE SALIENCES: CONTROLS > SCRABBLE EXPERTS**							
4	−63	−15	36	3.5058	21	Left Postcentral Gyrus	4
5	−60	−12	39	4.1222	44	Left Postcentral Gyrus	6
2	−9	60	18	3.8763	12	Left Superior Medial Gyrus	10
2	−69	−24	0	4.0236	34	Left Middle Temporal Gyrus	21
4	−12	−54	−6	3.6187	38	Left Lingual Gyrus	19
**RIGHT LATERALIZED POSITIVE SALIENCES: CONTROLS > SCRABBLE EXPERTS**							
5	36	−36	66	3.533	21	Right Postcentral Gyrus	4
5	42	6	−30	4.2324	31	Right Medial Temporal Pole	38
4	36	12	−6	3.7879	16	Right Insular Lobe	
2	6	−27	18	3.77	20	Right Thalamus	
5	9	−48	66	5.245	75	Right Precuneus	7
5	6	−93	21	5.4062	45	Right Cuneus	18
**LEFT LATERALIZED NEGATIVE SALIENCES: SCRABBLE EXPERTS > CONTROLS**							
3	−30	33	48	−4.1232	99	Left Middle Frontal Gyrus	8
4	0	54	39	−3.313	10	Left Superior Medial Frontal Gyrus	8
5	0	3	54	−3.3062	19	Left Posterior Medial Frontal Gyrus	6
3	−3	51	−18	−3.304	11	Left Rectal Gyrus	9
1	−48	6	30	−3.9804	101	Left Precentral Gyrus	6
3	−3	39	24	−3.7931	95	Left Anterior Cingulate Cortex	32
3	−18	−15	−21	−3.9978	31	Left ParaHippocampal Gyrus	28
3	−51	−51	21	−4.9044	67	Left Middle Temporal Gyrus	22
5	−42	−63	0	−5.1922	63	Left Inferior Temporal Gyrus	19
5	−27	−60	42	−3.731	15	Left Inferior Parietal Lobule	40
3	−60	−33	36	−5.2273	26	Left SupraMarginal Gyrus	40
3	−24	−15	15	−4.6959	57	Left Thalamus	
5	−42	−45	−18	−4.9583	82	Left Fusiform Gyrus	37
4	−30	−75	33	−4.065	23	Left Superior Occipital Gyrus	19
2	−30	−75	21	−4.0955	133	Left Middle Occipital Gyrus	19
4	−36	−90	−15	−4.5053	72	Left Inferior Occipital Gyrus	19
2	−6	−78	−9	−3.4307	14	Left Lingual Gyrus	18
3	−6	−48	42	−4.5197	97	Left Precuneus	7
**RIGHT LATERALIZED NEGATIVE SALIENCES: SCRABBLE EXPERTS > CONTROLS**							
3	24	30	57	−5.1065	67	Right Superior Frontal Gyrus	8
3	30	51	27	−4.513	147	Right Middle Frontal Gyrus	9
3	51	33	0	−3.277	17	Right Inferior Frontal Gyrus (pars Triangularis)	45
5	9	15	48	−2.9668	10	Right Posterior Medial Frontal Gyrus	6
4	48	9	33	−4.6566	41	Right Precentral Gyrus	6
3	9	−24	39	−4.3541	42	Right Middle Cingulate Cortex	31
4	18	−21	18	−3.5062	11	Right Thalamus	
3	24	0	−18	−3.0827	11	Right Amygdala	
3	33	6	−42	−4.3245	39	Right Medial Temporal Pole	38
3	54	12	−15	−4.1317	39	Right Temporal Pole	21
3	66	−24	3	−5.0417	65	Right Superior Temporal Gyrus	22
4	51	−27	−15	−3.8174	85	Right Inferior Temporal Gyrus	20
5	30	−54	45	−3.808	60	Right Angular Gyrus	40
3	57	−30	36	−5.9044	197	Right SupraMarginal Gyrus	40
2	30	−78	21	−4.286	26	Right Superior Occipital Gyrus	19
5	30	−87	0	−5.3187	298	Right Middle Occipital Gyrus	18
2	30	−78	−6	−3.8472	33	Right Fusiform Gyrus	19
4	12	−99	−9	−6.4586	50	Right Lingual Gyrus	18

#### fMRI analysis 2 two-group data-driven behavior PLS examining brain-anagramming score correlations in SDT

**Figure 5:**
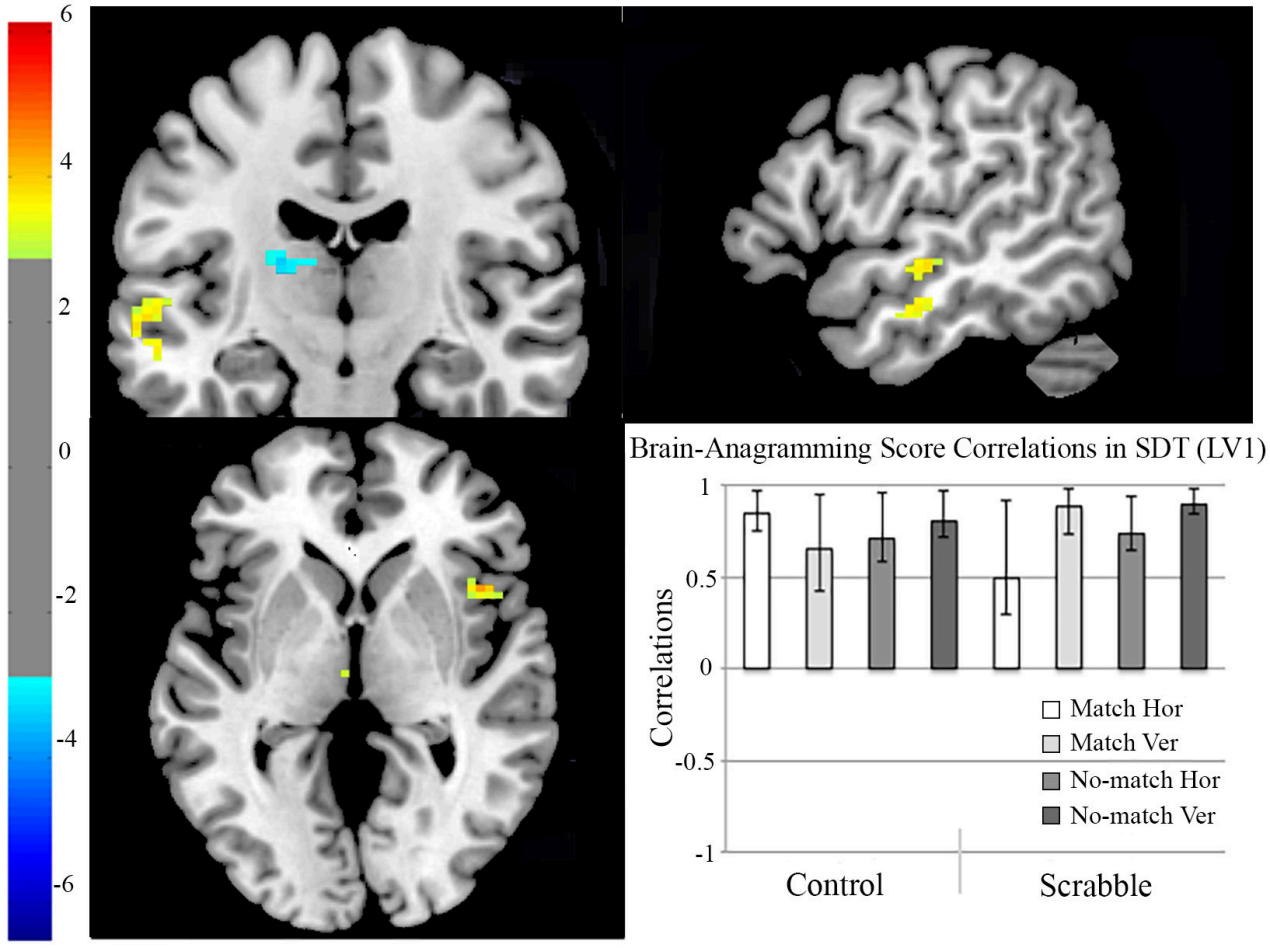
**fMRI analysis 2, two-group data-driven behavior PLS examining brain-anagramming score correlations in SDT, LV1**. On the brain images we illustrate brain-anagramming score correlations during the SDT. The brain is displayed in 3-plane view according to neurological convention (L = L). Regions highlighted in yellow indicate a positive correlation between higher anagramming scores and increased activation during the SDT for both groups. Regions highlighted in blue indicate a negative correlation between higher anagramming scores and increased activation in the SDT for both groups. The correlation bar graph captures the group and condition-related similarities in brain-anagramming score correlations during the SDT. Hor, horizontal presentation; Ver, vertical presentation; SDT, Symbol Decision Task; LV, Latent Variable.

To more directly test the link between Scrabble expertise and neural correlates of the SDT, we used the fMRI data to identify brain activity in relation to individual differences in Scrabble expertise (anagramming scores). We examined whether Scrabble experts and controls engaged different regions in the SDT that correlated with individual differences in anagramming scores. We identified two significant LVs. The first LV (*p* < 0.002, see Figure 5) highlighted brain-behavior correlations common to all conditions in both groups. Dominant positive saliences (highlighting regions where increased activity correlated positively with anagramming scores) included the right temporal pole and precentral gyrus, as well as the left superior medial temporal gyrus and mid portion of the middle temporal gyrus. Dominant negative saliences (highlighting regions where increased activity correlated negatively with anagramming scores) included the bilateral inferior parietal lobule, superior portion of the middle temporal gyri, superior temporal gyri, and precuneus, the right temporal pole, inferior frontal (pars orbitalis) and inferior temporal gyri, as well as the left fusiform gyrus (see Table 3 for full list of regions).

**Table 3:** **fMRI Analysis 2, Two-Group Behavior PLS Examining Brain-Anagramming Score Correlations in SDT, LV1 (Group Similarities)**.

**Lag**	**x**	**y**	**z**	**BSR**	**Cluster size**	**Region**	**BA**
**LEFT LATERALIZED POSITIVE SALIENCES: POSITIVE CORRELATION WITH ANAGRAMMING SCORES**							
1	−6	60	3	4.142	16	Left Superior Medial Frontal Gyrus	10
3	−60	−15	−15	3.697	12	Left Middle Temporal Gyrus	21
**RIGHT LATERALIZED POSITIVE SALIENCES: POSITIVE CORRELATION WITH ANAGRAMMING SCORES**							
3	36	−27	63	5.0442	24	Right Precentral Gyrus	3
4	36	18	−30	5.0571	29	Right Temporal Pole	38
1	27	18	−36	5.6429	28	Right Medial Temporal Pole	38
**LEFT LATERALIZED NEGATIVE SALIENCES: NEGATIVE CORRELATION WITH ANAGRAMMING SCORES**							
2	0	54	12	−3.7182	19	Left Superior Medial Frontal Gyrus	47
5	−36	51	12	−4.6114	11	Left Middle Frontal Gyrus	46
5	−48	−9	42	−5.0667	29	Left Postcentral Gyrus	4
2	−27	−12	12	−5.0822	42	Left Putamen	
5	−21	−3	21	−5.2532	19	Left Thalamus	
2	−51	−24	6	−4.3434	27	Left Superior Temporal Gyrus	41
2	−60	−18	−3	−6.1092	65	Left Middle Temporal Gyrus	22
5	−39	−42	54	−6.2014	165	Left Inferior Parietal Lobule	5
1	−21	−51	−15	−6.0734	204	Left Fusiform Gyrus	19
2	−30	−99	0	−4.0697	25	Left Middle Occipital Gyrus	18
5	−15	−93	−3	−4.6311	88	Left Calcarine Gyrus	17
5	−24	−51	3	−5.291	52	Left Precuneus	7
**LEFT LATERALIZED NEGATIVE SALIENCES: NEGATIVE CORRELATION WITH ANAGRAMMING SCORES**							
2	18	33	45	−4.2868	35	Right Superior Frontal Gyrus	8
5	9	39	36	−5.3957	28	Right Superior Medial Gyrus	8
2	27	57	6	−4.1304	20	Right Middle Frontal Gyrus	10
1	42	−6	18	−5.5238	27	Right Rolandic Operculum	4
2	45	45	−12	−4.2818	19	Right Inferior Frontal Gyrus (pars Orbitalis)	47
2	42	−12	39	−4.0526	17	Right Precentral Gyrus	4
1	48	−27	63	−4.7266	26	Right Postcentral Gyrus	3
2	6	−24	6	−6.5229	155	Right Thalamus	
5	18	−3	21	−5.3753	55	Right Caudate	
5	33	6	−42	−4.1423	20	Right Medial Temporal Pole	38
2	54	−9	−9	−5.0373	60	Right Superior Temporal Gyrus	21
3	51	0	−18	−6.1179	56	Right Middle Temporal Gyrus	21
1	60	−48	39	−3.9306	72	Right Inferior Parietal Lobule	40
4	27	−54	54	−3.9378	12	Right Inferior Parietal Lobule	7
5	9	−48	27	−5.0102	250	Right Posterior Cingulate Cortex	7
5	48	−75	−9	−5.0352	52	Right Inferior Occipital Gyrus	19
5	15	−96	−9	−3.9878	18	Right Lingual Gyrus	18
4	15	−75	36	−3.956	10	Right Cuneus	19
5	6	−66	51	−4.2025	14	Right Precuneus	7

**Figure 6:**
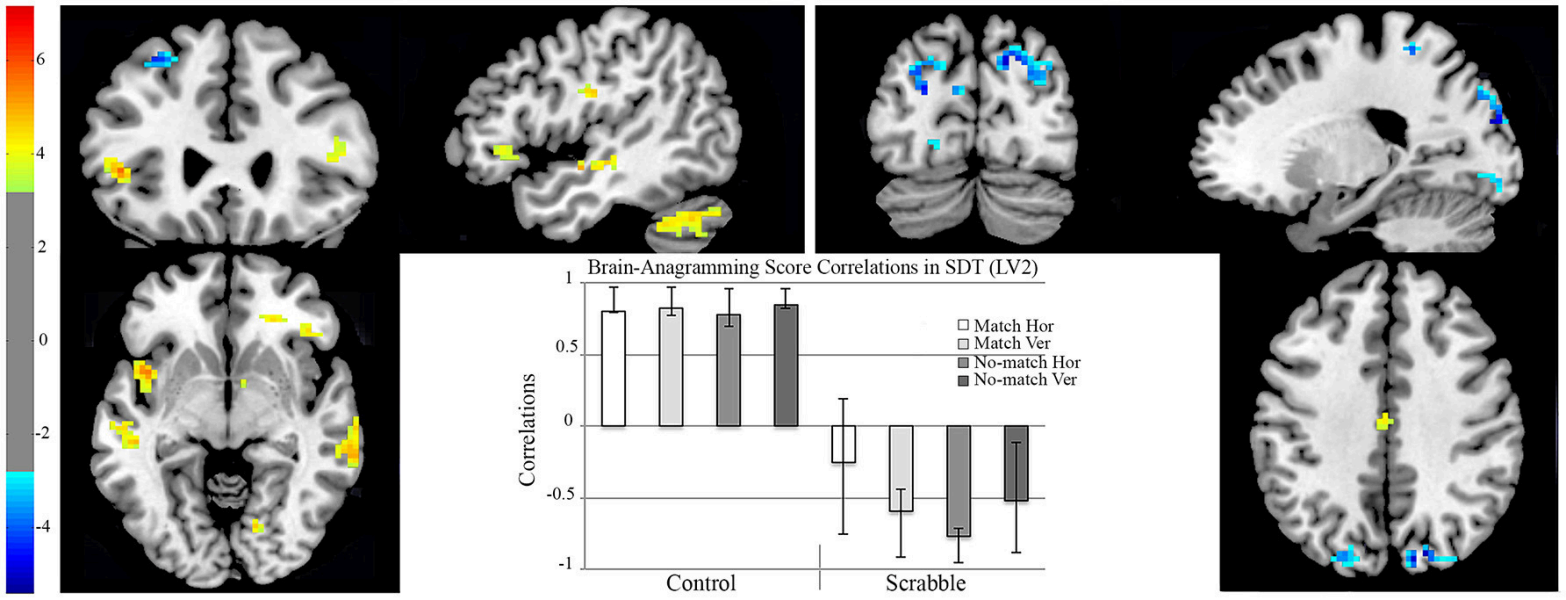
**fMRI analysis 2, two-group data-driven behavior PLS examining brain-anagramming score correlations in SDT, LV2**. On the brain images we illustrate brain-anagramming score correlations during the SDT. The brain is displayed in 3-plane view according to neurological convention (L = L). Regions highlighted in yellow indicate a positive correlation between higher anagramming scores and increased activation across all task conditions for controls. Regions highlighted in blue indicate a positive correlation between higher anagramming scores and increased activation during horizontal no-match and vertical match/no-match for Scrabble experts. The correlation bar graph captures the condition-dependent correlations between our behavior measure (anagramming scores) and the regions identified in the brain images. Hor, horizontal presentation; Ver, vertical presentation; SDT, Symbol Decision Task; LV, Latent Variable.

The second LV (*p* < 0.002, see Figure 6) identified group differences in regions engaged in the SDT that correlated with anagramming scores. Dominant positive saliences (highlighting regions in which increased SDT activity correlated with anagramming scores during all task conditions for controls) included the left inferior frontal (pars orbitalis) gyrus, the right superior temporal and fusiform gyri, as well as bilateral middle temporal gyri and temporal pole. Dominant negative saliences (highlighting regions in which increased activity correlated with anagramming scores for Scrabble experts during vertical match, horizontal no-match, and vertical no-match conditions) included the left fusiform and middle frontal gyri, the right angular, and inferior frontal (pars orbitalis) gyri, insula lobe, thalamus, and precuneus, as well as bilateral superior parietal lobule (see Table 4 for full list of regions).

**Table 4:** **fMRI Analysis 2, Two-Group Behavior PLS Examining Brain-Anagramming Score Correlations in SDT, LV2 (Group Differences)**.

**Lag**	**x**	**y**	**z**	**BSR**	**Cluster size**	**Region**	**BA**
**LEFT LATERALIZED POSITIVE SALIENCES: POSITIVE CORRELATION WITH ANAGRAMMING SCORES FOR CONTROLS**							
4	−24	−6	60	4.0182	19	Left Superior Frontal Gyrus	6
4	−42	27	−3	5.1115	68	Left Inferior Frontal Gyrus (pars Orbitalis)	45
4	−51	−12	36	5.1609	30	Left Postcentral Gyrus	1
3	−30	9	−30	4.4686	28	Left Temporal Pole	28
4	−21	3	−39	4.2199	72	Left Parahippocampal Gyrus	36
5	−3	−6	36	4.4782	13	Left Middle Cingulate Cortex	31
4	−51	−12	−9	4.4751	24	Left Middle Temporal Gyrus	22
4	−3	−72	3	4.1624	17	Left Lingual Gyrus	17
**RIGHT LATERALIZED POSITIVE SALIENCES: POSITIVE CORRELATION WITH ANAGRAMMING SCORES FOR CONTROLS**							
3	18	42	39	4.4915	10	Right Superior Frontal Gyrus	8
4	42	51	−6	3.9516	18	Right Middle Frontal Gyrus	46
2	42	15	−33	3.8667	16	Right Temporal Pole	21
4	36	−18	−18	4.2544	30	Right Hippocampus	
4	51	0	−12	5.0024	19	Right Superior Temporal Gyrus	22
4	66	−15	−12	4.8356	97	Right Middle Temporal Gyrus	21
2	39	−6	−33	4.5227	10	Right Fusiform	20
**LEFT LATERALIZED NEGATIVE SALIENCES: POSITIVE CORRELATION WITH ANAGRAMMING SCORES FOR SCRABBLE EXPERTS**							
4	−24	30	60	−4.3964	16	Left Middle Frontal Gyrus	6
5	−27	−45	66	−5.0819	26	Left Superior Parietal Lobule	7
5	−18	−84	24	−5.4013	69	Left Superior Occipital Gyrus	19
1	−42	−48	−24	−4.3672	15	Left Fusiform Gyrus	18
**RIGHT LATERALIZED NEGATIVE SALIENCES: POSITIVE CORRELATION WITH ANAGRAMMING SCORES FOR SCRABBLE EXPERTS**							
1	39	24	−24	−4.2576	13	Right Inferior Frontal Gyrus (pars Orbitalis)	45
3	39	−9	3	−4.3472	10	Right Insular Lobe	
1	18	−27	−3	−4.4839	16	Right Thalamus	
4	−18	−69	57	−4.4537	59	Right Superior Parietal Lobule	7
4	36	−78	42	−4.2231	47	Right Angular Gyrus	7
1	18	−78	45	−4.583	42	Right Cuneus	19
5	18	−75	45	−5.0716	99	Right Precuneus	7

#### fMRI analysis 3 two-group hypothesis-driven behavior PLS examining brain-anagramming score correlations in LDT and SDT

**Figure 7:**
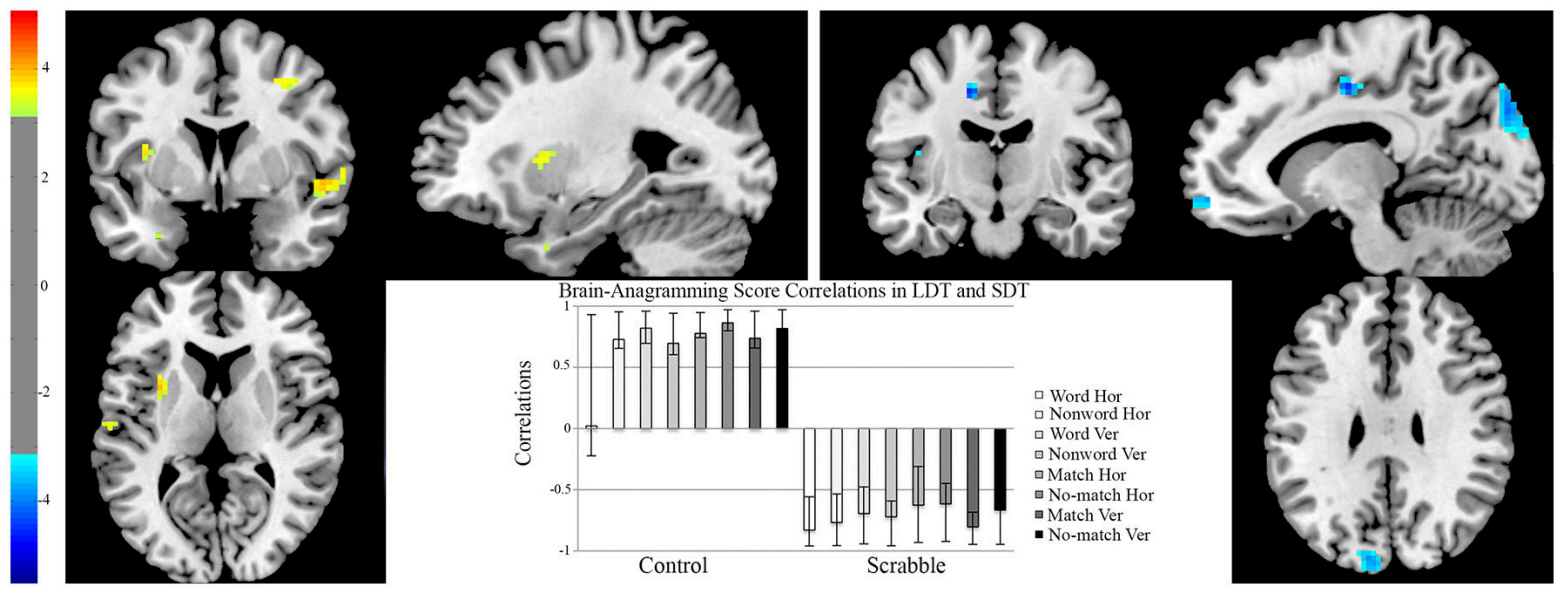
**fMRI analysis 3, two-group data-driven behavior PLS examining brain-anagramming score correlations in LDT and SDT**. On the brain images we illustrate brain-anagramming score correlations during the LDT and SDT. The brain is displayed in 3-plane view according to neurological convention (L = L). Regions highlighted in yellow indicate a positive correlation between higher anagramming scores and increased activation across horizontal non-words, vertical words/non-words, and all conditions of the SDT for controls. Regions highlighted in blue indicate a positive correlation between higher anagramming scores and increased activation across all conditions of the LDT and SDT. The correlation bar graph captures group differences in brain-anagramming score correlations during the LDT and SDT. Hor, horizontal presentation; Ver, vertical presentation; LDT, Lexical Decision Task; SDT, Symbol Decision Task.

To ensure that regions identified in analysis 2 for Scrabble experts overlapped with visual-perceptual and long-term working memory regions employed by that group for LDT performance, we performed a non-rotated behavior PLS, to examine if the group differences in brain-behavior correlations were common across both tasks. The group difference was significant (*p* = 0.01, see Figure 7). Positive saliences (regions where increased activity correlated with anagramming scores for controls for horizontal non-words, vertical words, vertical non-words and all SDT conditions) included the left middle cingulate gyrus and lingual gyrus, the right superior frontal gyrus and bilateral insular lobes. Negative saliences (regions where increased activity correlated with anagramming scores for Scrabble experts for all LDT and SDT task conditions) included the left middle frontal gyrus, thalamus, postcentral gyrus, middle temporal gyrus, fusiform gyrus, and cuneus, right inferior frontal gyrus, and bilateral superior frontal gyrus, superior temporal gyrus, and precuneus (see Table 5 for a full list of regions).

**Figure 8:**
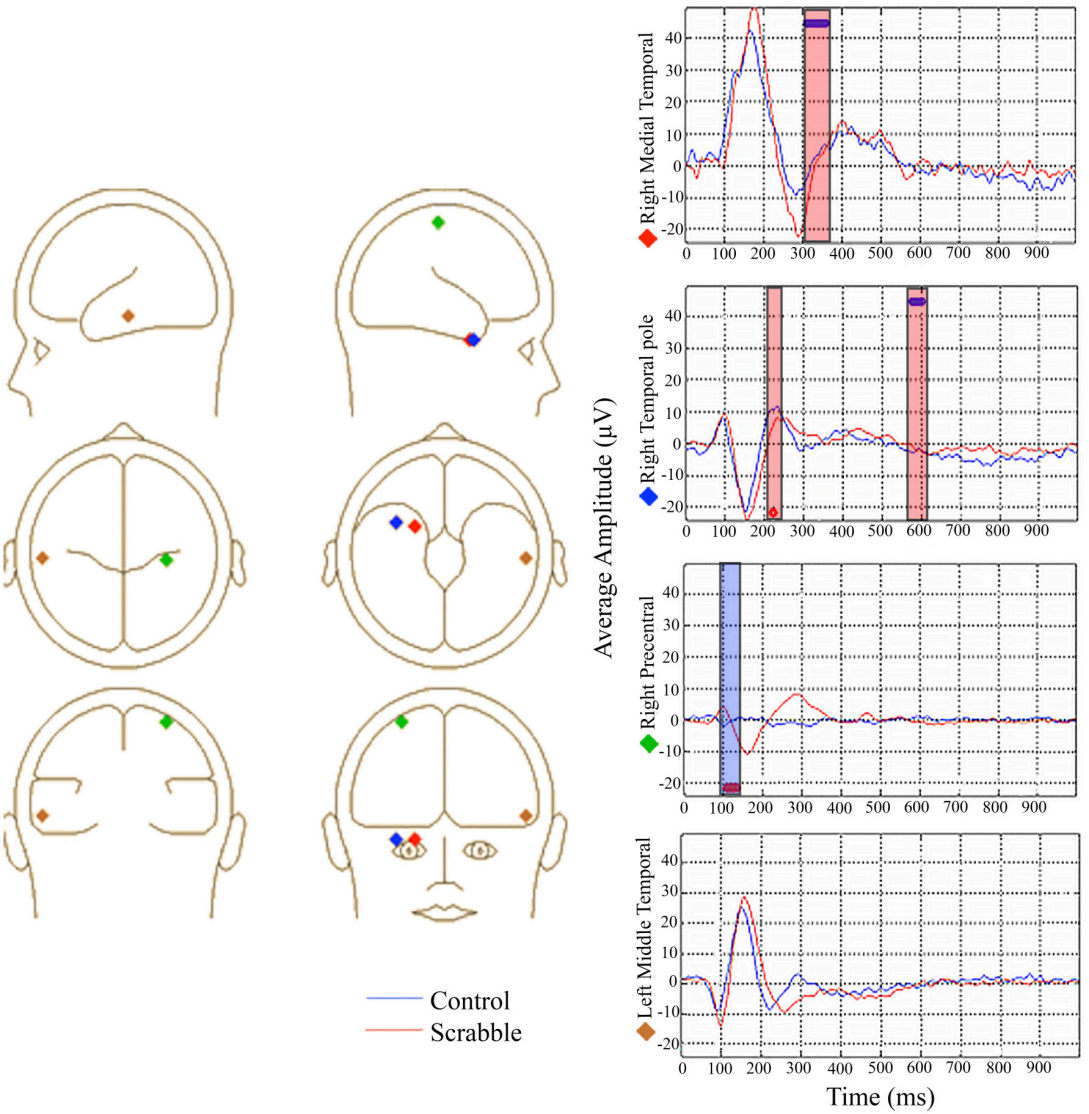
**ERP source model analysis 1, data-driven behavior PLS examining brain-anagramming score correlations in SDT, showing group similarities (blue circles above waveforms) and differences (red circles below waveforms) in source waveform-anagramming score correlations**. The locations of all sources are displayed on the left and average waveforms from sources of interest are displayed on the right. Blue boxes represent time windows where higher anagramming scores were associated with smaller amplitudes, and red boxes represent time windows where higher anagramming scores were associated with greater amplitudes of source waveforms. Source analysis was performed on each condition separately, although average waveforms are displayed for ease of interpretation.

**Table 5:** **fMRI Analysis 3, Two-Group Non-rotated Behavior PLS Examining Brain-Anagramming Score Correlations in SDT and LDT**.

**Lag**	**x**	**y**	**z**	**BSR**	**Cluster size**	**Region**	**BA**
**LEFT LATERALIZED POSITIVE SALIENCES: POSITIVE CORRELATION WITH ANAGRAMMING SCORES FOR CONTROLS**							
4	−3	−3	36	4.4598	12	Left Middle Cingulate Gyrus	24
3	−36	−12	15	5.1106	20	Left Insular Lobe	
4	−6	−75	3	3.6385	27	Left Lingual Gyrus	17
**RIGHT LATERALIZED POSITIVE SALIENCES: POSITIVE CORRELATION WITH ANAGRAMMING SCORES FOR CONTROLS**							
4	6	−15	66	3.6739	16	Right Superior Frontal Gyrus	6
4	48	0	−6	3.6087	14	Right Insula Lobe	
**LEFT LATERALIZED NEGATIVE SALIENCES: POSITIVE CORRELATION WITH ANAGRAMMING SCORES FOR SCRABBLE EXPERTS**							
5	−15	60	21	−3.8128	14	Left Superior Frontal Gyrus	9
2	−12	57	−9	−3.6242	12	Left Superior Medial Frontal Gyrus	10
5	−30	36	48	−4.2834	42	Left Middle Frontal Gyrus	6
5	−18	−15	15	−4.1959	10	Left Thalamus	
3	−12	30	27	−4.3575	15	Left Anterior Cingulate Cortex	32
2	−12	−12	45	−4.4292	14	Left Middle Cingulate Cortex	24
2	−42	−6	12	−4.4167	24	Postcentral gyrus	43
2	−69	−33	9	−4.0556	12	Left Superior Temporal Gyrus	22
3	−54	6	−27	−5.0967	25	Left Middle Temporal Gyrus	21
4	−15	−72	60	−3.5152	12	Left Superior Parietal Lobule	7
5	−45	−45	−24	−5.5405	71	Left Fusiform Gyrus	36
2	−12	−90	33	−3.9408	56	Left Cuneus	19
5	−15	−72	33	−4.6092	30	Left Precuneus	19
**RIGHT LATERALIZED NEGATIVE SALIENCES: POSITIVE CORRELATION WITH ANAGRAMMING SCORES FOR SCRABBLE EXPERTS**							
5	24	60	15	−5.5653	82	Right Superior Frontal Gyrus	10
3	30	24	12	−5.3679	26	Right Inferior Frontal Gyrus	45
2	57	−48	15	−3.9568	17	Right Superior Temporal Gyrus	22
5	27	−81	21	−4.9811	28	Right Superior Occipital Gyrus	19
5	21	−75	39	−4.6051	64	Right Precuneus	7

### ERP results

#### ERP source model analysis 1 two-group data-driven behavior PLS examining brain-anagramming score correlations in SDT

The common regions across groups/conditions, identified in LV1 from fMRI Analysis 2 were used to guide our regions of interest for source analysis. The model was created by first fitting the source waveform from the right medial temporal pole, followed by the right precentral gyrus, the left middle temporal gyrus, and the right temporal pole. This model accounted for an average of 73% of the variance across groups/conditions (range 68–77%). The purpose of this source model was to examine the time course of activity specifically in the regions identified through fMRI as commonly supporting SDT performance and correlating with anagramming scores. Its purpose was *not* to select a set of regions that explain as much of the variance in the data as possible. Thus, we did not attempt to alter the model to increase explained variance. Source waveforms were extracted from these four regions for each participant and analyzed using behavior PLS. Anagramming scores were used as the behavioral measure to examine whether there were group and/or condition-dependent differences in the timing/amplitude of source waveforms related to Scrabble expertise (see Lobaugh et al., 2005 for similar analyses).

**Figure 9:**
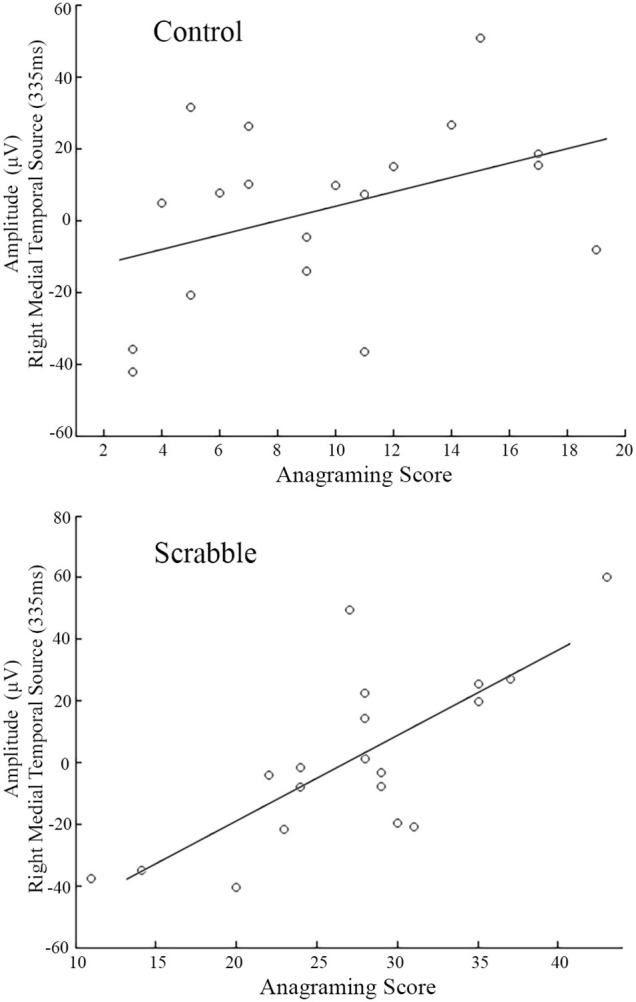
**ERP source model analysis 1, amplitude-anagram score correlations for controls (top) and Scrabble experts (bottom) in the right medial temporal source waveform at 335 ms**. Amplitudes are averaged across SDT conditions for each participant and linear fits are also plotted for each group.

We identified two significant LVs. For the first LV (*p* < 0.001), the anagram-source waveform correlations were equally strong in all conditions across groups, indicating similarities in timing/amplitude of source waveforms. For the right medial temporal pole source waveform, participants with higher anagramming scores had larger positive amplitudes in the time window of the P300 component (310–360 ms) (see Figure 9). For the right temporal pole source waveform, participants with higher anagramming scores had larger positive amplitudes in the time window of the P600 component (575–600 ms). No significant results were found in the right precentral or left MTG source waveforms.

The second LV identified a group difference (*p* < 0.001). This LV was dominated by the time course of neural activity for control participants (i.e., the confidence intervals of all task conditions crossed 0 for Scrabble experts, see Figure 5). For the right temporal pole, participants with higher anagramming scores had larger positive amplitudes in the time window of the P200 component (215–225 ms). For the right precentral gyrus, participants with higher anagramming scores had smaller negative amplitudes in the time window of the N100 component (105–135 ms). There was a large amount of variability in the right precentral source waveform in the control group, which lead to an average waveform with relatively low amplitudes (see Figure 8).

#### ERP source model analyses 2 and 3 separate data-driven behavior PLSs examining brain-anagramming score correlations in SDT for control group and for scrabble expert group

As the fMRI analysis also identified group differences in LV2, we investigated the source waveforms from regions activated across conditions for each group separately using the same method of source waveform modeling as described above for LV1. For the control group, a network was identified across all four conditions. From these results, the first region that fit into the source waveform model was the left lingual gyrus, followed by the right fusiform gyrus, the left mid cingulate cortex, and the left middle temporal gyrus. This model accounted for an average of 90% of the variance (range 89–91%) across conditions. One significant LV was identified (*p* = 0.008) where anagram-source waveform correlations were negative for all four conditions, indicating similarities in timing/amplitude of source waveforms across conditions. For the left MTG source waveform, participants with higher anagramming scores had larger positive amplitudes in the time window of the P300 component (325–340 ms) and the P600 component (525–535 ms). For the left lingual gyrus source waveform, participants with higher anagramming scores had smaller negative amplitudes in the time window of the N200 component (195–230 ms).

**Figure 10:**
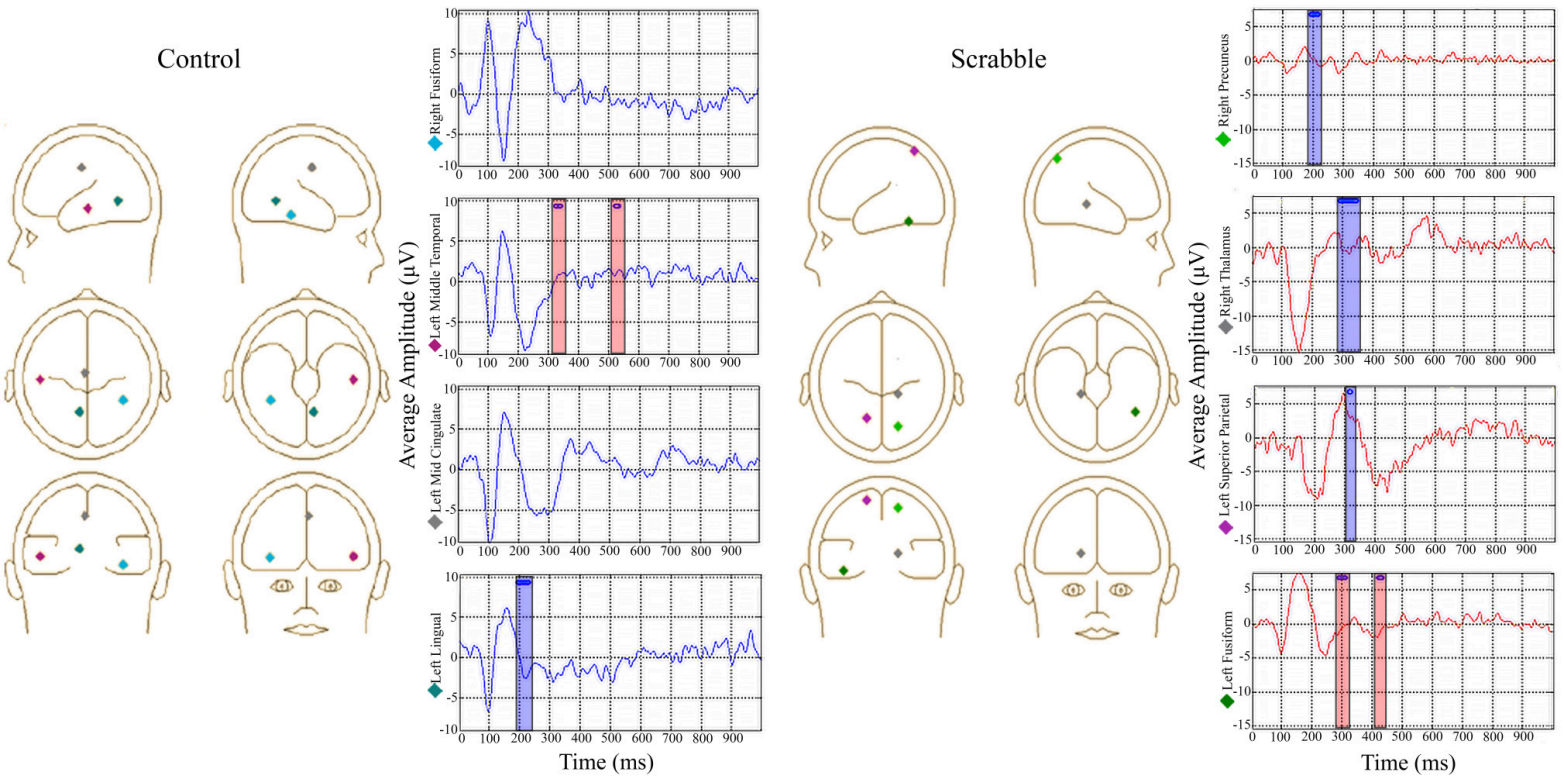
**ERP source model Analysis 2 and 3, data-driven behavior PLS examining brain-anagramming score correlations in SDT, showing source waveform-anagramming score correlations for each group**. The locations of all sources are displayed on the left and average waveforms from sources of interest are displayed on the right. Blue boxes represent time windows where higher anagramming scores were associated with smaller amplitude, and red boxes represent time windows where higher anagramming scores were associated with greater amplitudes of source waveforms. Source analysis was performed on each condition separately, although average waveforms are displayed for ease of interpretation.

For the Scrabble group, we identified common regions activated across vertical match, horizontal no-match, and vertical no-match conditions. From these results, the first region that fit into the source waveform model was the left superior parietal lobe, followed by the left fusiform gyrus, the right thalamus, and the right middle temporal gyrus. This model accounted for an average of 84% of the variance (range 81–87%) across conditions. We identified one significant LV (*p* < 0.001), where anagram-source waveform correlations were negative for all three conditions. For the right precuneus source waveform, participants with higher anagramming scores had smaller negative amplitudes in the time window of the N200 component (195–215 ms). For the right thalamus source waveform, participants with higher anagramming scores had smaller positive amplitudes in the time window of the P300 component (290–345 ms). For the left superior parietal lobule source waveform, participants with higher anagramming scores had smaller positive amplitudes in the time window of the P300 component (315–320 ms). For the left fusiform gyrus source waveform, participants with higher anagramming scores had larger positive amplitudes in the time window of the P300 component (290–315 ms), and larger negative amplitudes in the time window of the N400 component (420–435 ms) (see Figure 10).

## Discussion

The purpose of the current study was to test the limits of transfer for competitive Scrabble expertise. Previous studies identified *near* transfer through enhanced performance for competitive Scrabble players on a visual LDT compared to age-matched controls (Halpern and Wai, 2007; Hargreaves et al., 2012; Protzner et al., 2016). In LDT, Scrabble experts showed decreased reliance on word meaning as well as greater vertical fluency (Hargreaves et al., 2012). The aim of the present study was to investigate *far* transfer of Scrabble-related expertise to the SDT. This could be observed in behavior, if vertical fluency for Scrabble experts transfers to non-letter symbols. It also could be observed in brain, if the regions associated with the SDT for Scrabble experts are different than those used by controls and similar to those engaged by Scrabble experts in LDT.

We found no differences between Scrabble experts and controls in their behavioral performance on the SDT, suggesting no behavioral transfer. That is, the enhanced performance of Scrabble experts seen for vertical words in LDT was not found for vertical stimuli in the SDT, suggesting that the vertical fluency developed by Scrabble experts is specific to letter stimuli. Despite the lack of behavioral transfer, our behavioral results confirm that the similar aspects of the SDT and LDT (i.e., the presentation of both horizontally—and vertically-presented visual strings, and the requirement for binary decisions) have consistent RT outcomes. That is, in both tasks, participants were faster and more accurate making decisions for horizontal strings, and for “yes” (match) responses. Horizontal fluency and lexicality effects are typically observed in LDT (e.g., Hargreaves et al., 2012), and variants of these effects were present in the SDT.

Several previous studies in language and other domains have identified differences between the neural networks activated across groups in the absence of differences in task performance (e.g., Bennett et al., 2001; Grady et al., 2010; Hargreaves et al., 2011; McIntosh et al., 2014). In the context of expertise, Maguire et al. (2003) examined expert memorisers and non-expert controls, and reported group differences in the neural regions engaged during both familiar and unfamiliar memory tasks. However, engaging these brain regions during the unfamiliar task did not result in a behavioral advantage for the expert memorisers. These results suggest that experts developed a task strategy that was not available to the controls, and, despite no behavioral benefit, experts employed this strategy in an unfamiliar context. Thus, it is possible that the different neural mechanisms engaged by Scrabble experts during a lexical task, such as visual perceptual and long-term working memory regions, may also be engaged in an unfamiliar non-lexical task that shares similar processing components. As such, we next examined whether Scrabble experts would show far transfer in brain by engaging different neural mechanisms compared to controls during SDT performance.

Our fMRI results showed a significant difference between groups in the regions engaged during the SDT. Specifically, regions engaged by Scrabble experts in the SDT were more widespread, including extensive activity in posterior visual regions, as well as temporal and parietal language regions and their right hemisphere homologs. These results align with the expertise literature, with respect to recruitment of additional regions as well as greater bilateral activity in expert groups (Bilalić et al., 2011, 2012; Guida et al., 2012; Proverbio et al., 2013). In contrast, the control group engaged fewer regions, with activity found predominantly in temporal and frontal regions. These findings suggest that Scrabble experts and controls use different neural substrates to support SDT performance, but the findings do not link differences specifically to Scrabble expertise.

To link brain changes directly to Scrabble expertise, we constrained our analyses to show only those brain regions in which changes in activity were associated with a measure of Scrabble expertise: anagramming scores. This analysis identified both commonalities and differences between groups. Commonalities among regions (i.e., regions that were associated with individual differences in anagramming that did not vary by group or task condition) included the left middle temporal and medial frontal gyri, as well as the right precentral gyrus and temporal pole. Thus, our results suggest that for both groups, higher anagramming scores were associated with greater reliance on these regions regardless of orientation or whether stimuli contained matching symbols. The EEG data provided additional insights about the time course of processing in these common regions. For both Scrabble experts and controls, higher anagramming scores were correlated with increased amplitude of the P300 component in the right medial temporal source waveform. The P300 typically peaks around 300 ms post-stimulus at centro-parietal electrodes with neural generators in temporal and parietal regions (Polich, 2007; Dong et al., 2015), consistent with the timing and location of our source locations. This component has been associated with working memory and attentional processing (Patel and Azzam, 2005; Polich, 2007; Portella et al., 2012), and greater P300 amplitudes have been found to correlate positively with working memory capacity (Dong et al., 2015). Interestingly, this finding suggests that regardless of group membership (i.e., expert or control), participants with greater anagramming scores may have greater long-term working memory capacity in the SDT, as evident by greater P300 amplitudes in the right medial temporal lobe. This is consistent with Tuffiash et al. (2007) anagrammatic word-identification skill hypothesis, which proposes anagramming skill to affect domain-specific long-term working memory, although in our case, it applies to all participants with greater anagramming skills, not exclusively to Scrabble experts. However, given the heterogeneity of the timing of the P300 across tasks and modalities in the literature, an alternative explanation is that larger P300 amplitudes may reflect less variability in the participants who are performing the tasks more efficiently.

Importantly, we also identified group differences in the regions engaged during the SDT related to anagramming scores, suggesting far transfer of Scrabble expertise to the SDT in the context of our brain data. Controls with higher anagramming scores engaged more left hemisphere language regions and right hemisphere language homologs, whereas the regions engaged for Scrabble experts with higher anagramming scores included the precuneus, superior parietal lobule, and insula. The EEG analyses within these regions again showed increased amplitude of the P300 to correlate with higher anagramming scores, although this time, in different locations. For the control group this result was found in the left middle temporal gyrus, a region typically associated with access to word meaning. For Scrabble experts this result was found more posteriorly in the left fusiform gyrus, a region associated with earlier orthographic analysis of visual word forms (Carreiras et al., 2014). This difference between groups may reflect greater reliance on earlier perceptual processing for Scrabble experts compared to controls.

To confirm that group differences truly represented far transfer in our fMRI data, we performed a direct comparison of the neural substrates that support near transfer during the LDT (Protzner et al., 2016), and the neural substrates identified here during the SDT for Scrabble experts and controls. We identified a significant group difference, where the regions associated with anagramming skill engaged by Scrabble experts included posterior visual areas and long-term working memory regions (e.g., superior occipital gyrus and superior parietal lobe), similar to those found in Protzner et al. (2016). The regions identified in controls included left posterior visual and right frontal regions. However, activity in these regions correlated with anagramming scores only for the relatively unfamiliar task conditions for the controls (symbols, non-words, and vertical words), and not for the highly familiar task condition (horizontal words). In contrast, anagramming scores correlated with activity across all task conditions of the LDT and SDT for Scrabble experts. These similarities suggest transfer in the neural mechanisms engaged in the two tasks by Scrabble experts. That is, Scrabble expertise changes the neural substrates associated with the performance of both the LDT (Protzner et al., 2016) and SDT in similar ways.

Competitive Scrabble players have extraordinary word recognition skills that provide near transfer benefits in LDT. Our data suggest that the behavioral benefits of Scrabble expertise are limited to tasks that bear a strong resemblance to the specific domain of expertise, and do not show far transfer to the SDT. However, for both near and far transfer tasks, Scrabble expertise alters neural substrates that support task performance. Considered together, our behavioral and brain findings paint a complex picture of the consequences of expertise, one that suggests that we may need to revise the traditional characterization of transfer as an all-or-nothing phenomenon. That is, the study of transfer should not only encompass the end product of task performance, but also consider the underlying mechanisms engaged. Although the Scrabble experts did not show enhanced behavioral performance in the SDT, the neuroimaging results provide evidence of functional reorganization that extends beyond linguistic processing. Such reorganization may have implications for our understanding of learning and the consequences of long-term training.

## Author contributions

PP and AP conceptualized the study. IH and LZ assisted with task design and data collection. SVH, JH, KMS, and FC contributed to data processing and analysis. SVH wrote manuscript. PP, AP assisted with writing the manuscript.

## Funding

This research was supported by a Harley N. Hotchkiss Postdoctoral Fellowship and Alberta Innovates Health Solutions Fellowship to SVH, Natural Sciences and Engineering Research Council of Canada (NSERC) Discovery Grants to AP [grant number 418454-2013] and PP [grant number 217309-2013], Canadian Foundation for Innovation Leaders Opportunity Fund to AP [grant number 30320], and Alberta Enterprise and Advanced Education Research Capacity Program, Alberta Alignment Grant to AP [grant number RCP-13-38-SEG].

### Conflict of interest statement

The authors declare that the research was conducted in the absence of any commercial or financial relationships that could be construed as a potential conflict of interest.
