# Supplementary material for: Testing the Limits of Skill Transfer for Scrabble Experts in Behavior and Brain
Source: Front Hum Neurosci. 2016 Nov 9;10:564. doi: 10.3389/fnhum.2016.00564 (PMC5101412; doi:10.3389/fnhum.2016.00564)
Supplement: Supplementary file 1 [file DataSheet1.docx]

***Supplementary Material***

**Testing the Limits of Skill Transfer for Scrabble Experts in Behaviour and Brain**

**Sophia van Hees*, Penny M. Pexman, Ian S. Hargreaves, Lenka Zdrazilova, Jessie M. Hart, Kaia Myers-Stewart, Filomeno Cortese, and Andrea B. Protzner**

*** Correspondence:** Sophia van Hees: svanhees@ucalgary.ca

# Supplementary Information on Partial Least Squares Analysis

**1.1 PLS for fMRI data**

**1.1.1 Task PLS (used for fMRI analysis 1):** Task PLS uses singular value decomposition to identify distributed activity patterns, or latent variables (LV), that show similarities or differences between participant groups and experimental conditions. The most commonly used version of task PLS is similar to principal components analysis, in that contrasts across conditions or groups typically are not specified in advance. Rather, the algorithm extracts LVs explaining the covariance between conditions and brain activity in order of the amount of covariance explained (with the LV accounting for the most covariance extracted first). We used a *nonrotated* version of task PLS, in which a priori contrasts restrict the patterns derived from task PLS (McIntosh & Lobaugh, 2004). The effect of interest was a main effect of group (Scrabble experts versus controls).

PLS operates on the entire data structure at once (i.e., all groups and conditions), which requires that the data be in matrix form. The rows of the data matrix are arranged as follows. Subject groups are stacked. Within group, condition blocks are stacked and each participant has a row of data within each condition block. With *g* groups, *n* participants and *k* conditions, there are *g* × *n* × *k* rows in the matrix. The columns of the data matrix contain the signal intensity measure at each voxel at each time point. The first column has intensity for the first voxel at the first time point, the second column has the intensity for the first voxel at the second time point. With *m* voxels and *t* time points, there are *m* × *t* columns in the matrix. The hemodynamic response function (HRF) for any given condition normally lasts for several scans; thus, a ‘‘lag window’’ is defined for as a short signal segment within a trial that represents the response of each voxel. In the current experiment, the lag-window size was 6 (TR = 2, 12 s), beginning at the onset of each trial. The HRF for each trial is expressed as the intensity difference from trial onset.

The effect of interest for our nonrotated task PLS was a main effect of group (Scrabble experts versus controls). PLS results are expressed in terms of latent variables (LVs), each of which contains three vectors. The first vector contains a *singular value*, which indicates the strength of the effect expressed by the LV. The remaining two vectors relate experimental design and brain activity. The experimental design vector contains *task saliences*, which indicate the degree to which each condition within each group is related to the brain signal pattern identified in the LV. These saliences can be interpreted as the contrast that codes the effect depicted in the LV. The brain signal vector contains *voxel saliences*. These are numerical voxel weights that identify the collection of voxels that, as a whole, are most related to the effects expressed in the LV. Note that for each LV, there is one salience per voxel that applies for all groups and all experimental conditions. Multiplying the BOLD signal value in each brain voxel for each subject by the salience for that voxel, and summing across all voxels, gives a ‘‘brain score’’ for each subject on a given LV. Brain scores indicate the degree to which each subject shows the spatial pattern of voxels expressed in the LV.

**1.1.2 Behaviour PLS (used for fMRI analyses 2 and 3):** Behaviour PLS examines group- and condition-dependent *correlations* between a behaviour measure (i.e., anagramming scores) and voxel signal intensity throughout the brain. We performed both data-driven and nonrotated behaviour PLS (as specified in Section 2.9: Image analyses, as well as in section 3.3: fMRI results) on a correlation matrix, comprised of the covariance between voxel signal and anagramming scores across participants in each group. We used the data driven version of behaviour PLS in contexts where we felt that that a more exploratory focus would be informative, and the nonrotated version where we wanted to test a specific a-priori hypothesis. Behaviour PLS results are very similar to those from task PLS, except latent variables show similarities or differences between groups and experimental conditions in terms of *brain-behaviour correlations*.

**1.1.3 Statistical assessment:** Statistical assessment was performed using permutation tests for the LVs and bootstrap estimates of standard errors for the voxel saliences. The permutation tests involved re-sampling with reassignment of the order of conditions for each subject (with the order of subjects remaining fixed) to assess whether the effect identified in a given LV is sufficiently strong to be differentiated from random noise. If the probability of obtaining a permuted singular value higher than the observed singular value was very low, the LV was considered to be significant. The bootstrap tests involved re-sampling with reassignment of subjects (with the order of conditions remaining fixed) to assess the reliability of non-zero voxel saliences within significant LVs. If the voxel salience is not greatly dependant on which subjects are included in the sample, then it is considered to be reliable. No corrections for multiple comparisons are necessary because the voxel saliences are calculated in a single mathematical step on the whole brain. The bootstrap ratio is proportional to a *z* score, but it should be interpreted as a confidence interval.

**2.2 PLS for EEG data**

**2.1.1 Behaviour PLS (used for ERP source model analyses 1, 2 and 3):** We performed both data-driven and nonrotated behaviour PLS (as specified in Section 2.9: Image analyses, as well as in section 3.4: EEG results) using anagramming scores, on the source waveforms from each SDT condition to identify similarities or differences in timing/amplitude of the source waveforms, associated with individual differences in anagramming scores, across groups/conditions. These analyses were very similar to those performed for the behavioural PLS for fMRI data, except LVs show similarities or differences between groups and experimental conditions in terms of source waveform-behaviour correlations.

For additional information about PLS’s application to event-related fMRI and ERP data, see McIntosh, Chau, and Protzner (2004) and Lobaugh, West and McIntosh (2001) respectively.
